# Microfluidics-Based Bioassays and Imaging of Plant Cells

**DOI:** 10.1093/pcp/pcab067

**Published:** 2021-07-01

**Authors:** Naoki Yanagisawa, Elena Kozgunova, Guido Grossmann, Anja Geitmann, Tetsuya Higashiyama

**Affiliations:** Institute of Transformative Bio-Molecules (ITbM), Nagoya University, Nagoya Furo-cho, Chikusa-ku, Nagoya, Aichi 464-8601, Japan; Department of Plant Biotechnology, Faculty of Biology, University of Freiburg, Freiburg, Schänzlestr. 1, Freiburg, Baden-Württemberg 79104, Germany; Institute of Cell and Interaction Biology, Heinrich Heine University Düsseldorf, Universitätsstr. 1, Düsseldorf 40225, Germany; Centre for Organismal Studies, Heidelberg University, Heidelberg, Baden-Württemberg 69120, Germany; Department of Plant Science, Faculty of Agricultural and Environmental Sciences, McGill University, Québec H9X 3V9, Canada; Institute of Transformative Bio-Molecules (ITbM), Nagoya University, Nagoya Furo-cho, Chikusa-ku, Nagoya, Aichi 464-8601, Japan; Division of Biological Science, Graduate School of Science, Nagoya University, Furo-cho, Chikusa-ku, Nagoya, Aichi 464-8602, Japan; Department of Biological Sciences, Graduate School of Science, The University of Tokyo, 7-3-1 Hongo, Bunkyo City, Tokyo 113-0033, Japan

**Keywords:** Microfluidics •, Moss protonemata •, Plant roots •, Pollen tubes •, Protoplasts •, Root hairs

## Abstract

Many plant processes occur in the context of and in interaction with a surrounding matrix such as soil (e.g. root growth and root–microbe interactions) or surrounding tissues (e.g. pollen tube growth through the pistil), making it difficult to study them with high-resolution optical microscopy. Over the past decade, microfabrication techniques have been developed to produce experimental systems that allow researchers to examine cell behavior in microstructured environments that mimic geometrical, physical and/or chemical aspects of the natural growth matrices and that cannot be generated using traditional agar plate assays. These microfabricated environments offer considerable design flexibility as well as the transparency required for high-resolution, light-based microscopy. In addition, microfluidic platforms have been used for various types of bioassays, including cellular force assays, chemoattraction assays and electrotropism assays. Here, we review the recent use of microfluidic devices to study plant cells and organs, including plant roots, root hairs, moss protonemata and pollen tubes. The increasing adoption of microfabrication techniques by the plant science community may transform our approaches to investigating how individual plant cells sense and respond to changes in the physical and chemical environment.

## Introduction

How are nutrients taken up by plant roots and transported toward and within the vasculature? What cellular and subcellular signaling and defense processes occur in plant cells during pathogen attack? How do plant cells and organs perceive, process and respond to dynamic environmental signals, obstacles or mechanical force? Answering such fundamental open questions in plant biology requires high-resolution imaging of living cells with precise control over the specimen’s microenvironment. Classical cell culture approaches with plants grown on agar surfaces usually do not allow dynamic control of environmental conditions, a deficiency that has been addressed by an increasing adoption of microfluidics in plant biology.

**Fig. 1:**
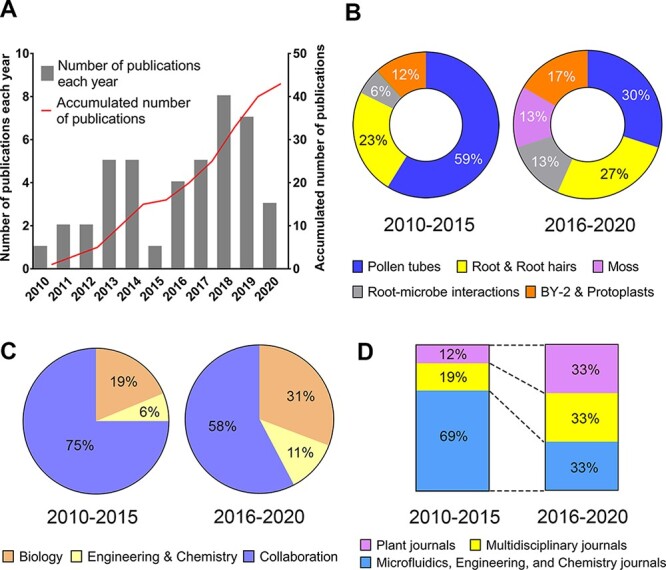
Trends in microfluidics-based research in plant cell biology. For this analysis, we used two search engines (Scopus and Web of Science) and keywords including microfluidic(s), microdevice, plants, pollen tubes, roots, root hairs, moss or protoplasts. (A) Number of original research papers published in 2010–2020. Review papers, conference proceedings, preprints and protocol papers based on the original research articles were not included in this survey. (B) Types of plant cells or organs studied using microfluidic techniques in 2010–2015 and 2016–2020. (C) Authors’ research disciplines (affiliations) categorized into Engineering & Chemistry, Biology or collaboration between these two fields. (D) Types of scientific journals publishing microfluidics-based research in plant cell biology. Digits after decimal point are removed.

Microfluidic devices are now widely used as experimental tools for various types of plant cell research. The ability to create micro-scale structures by photolithography and to manipulate the flow of liquid medium around the cells of interest has inspired many researchers to develop custom device–based cell growth chambers whose functions cannot be replaced by conventional approaches. The first ‘plant on a chip’ device, which was reported in 2010, allowed chemical stimulation of an *Arabidopsis thaliana* root at precisely defined regions (Meier et al. 2010). Another early study used microsystem design to study pollen tube guidance by isolated ovules (Yetisen et al. 2011). This pioneering work attracted considerable attention from the plant science community, prompting an increasing number of publications describing microfluidic techniques for analyzing plant cells or organs (**Fig. 1A**) over the past decade (2010–2020).

While the first devices were produced with channels and features that measured hundreds of micrometers, soon thereafter, the first microstructured device able to accommodate assays for single plant cells was developed (Agudelo et al. 2012). While pollen tubes, plant roots and protoplasts were the main subjects of research at the beginning of this period (Grossmann et al. 2011, Agudelo et al. 2013a, Zaban et al. 2014), microfluidics has more recently been employed for live imaging of the moss *Physcomitrella patens* (Sakai et al. 2019) (**Fig. 1B**). Within the experimental portfolio of plant biology, microfluidic devices have successfully populated the niche of microscale approaches (Clark et al. 2020). It is expected that these techniques will be used to investigate many more complex biological systems such as interactions between plant roots and soil microbes (Stanley et al. 2016, Poole 2017, Aleklett et al. 2018).

Strong collaborations between researchers in microfluidic engineering and plant biology have been crucial for the initial development of this multidisciplinary research field. In recent years, the technology has become increasingly accessible to biologists (**Fig. 1C**), and many labs have started designing, developing and operating such platforms to address questions that are difficult to answer using conventional experimental assays. Consequently, while the founding papers were often published in microfluidics journals, an increasing number of articles in this field have been published in plant research journals or multidisciplinary journals (**Fig. 1D**). The increasing use of microfluidic techniques by the plant biology community could be attributed to the ease of development of platforms using polydimethylsiloxane (PDMS) via a simple soft-lithography technique (Duffy et al. 1998). Other materials are also available for device preparation (Gale et al. 2018), but PDMS has proven ideal for plant cell studies due to its ease of fabrication, optical transparency, permeability to air and biocompatibility. Importantly, several detailed protocols have been published, explaining the fabrication and use of PDMS devices and, thus, helping new adopters of the technology to identify the right approach and the needed funds and infrastructure.

Reflecting the rapid development of this research field, several review papers have described the use of microfluidic techniques for live-cell imaging (Grossmann et al. 2018), examining root–bacteria interactions (Stanley et al. 2016, Aleklett et al. 2018), and a wide range of other applications (Sanati Nezhad 2014b, Elitaş et al. 2017) in plant cell research. In this review, we focus on advances in the development of microfluidic devices to analyze plant cells and organs over the past 5 years (2016–2020). We discuss microfluidics-assisted live-cell imaging techniques for plant roots, root hairs and moss cells, with an emphasis on microfluidic design as well as microflow-controlled environments for plant cell analysis. Next, we describe various microfluidics-based cellular assays that have been used to study pollen tubes, including a cellular force mechanical assay and chemotropism and electrotropism assays. A list of microfluidic devices developed for plant cell and organ research (years 2016–2020) is available in **supplementary data (Table S1)**. Also, we have, where applicable, highlighted the corresponding protocol following the mentioning of a particular device.

Finally, we provide perspectives on the roles of microfluidics in plant cell research and hitherto unexplored microfluidic techniques that have the potential to advance our understanding of plant cells at the molecular level in the future.

## Microfluidics-Assisted Plant Cell Imaging

### Plant roots and root hairs

**Fig. 2:**
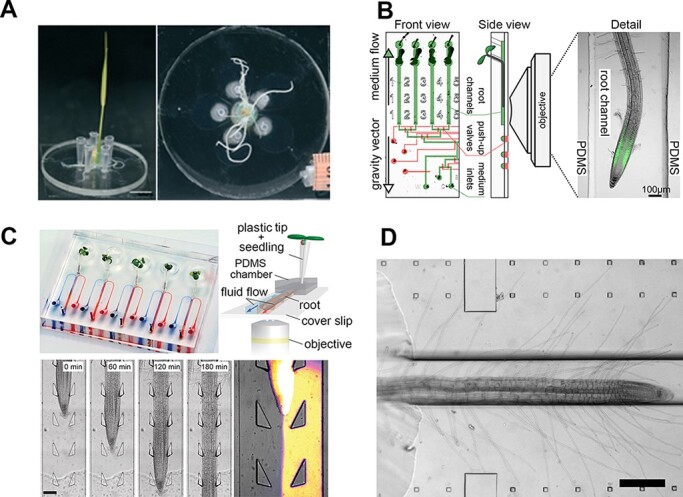
Microfluidics-based plant root growth chambers. (A) *Oryza sativa* root culture in the petaloid root growth chamber filled with liquid medium (after 9 day cultivation). Adapted from Chai et al. (2019) with permission from The Royal Society of Chemistry. (B) Vertically mounted RootChip, which allows *A.thaliana* root growth to be visualized under a confocal microscope. Reproduced from Fendrych et al. (2018) with permission from Springer Nature Ltd. (C) Dual-flow RootChip for *A. thaliana* roots growing through micro-fabricated pillars. This channel design allows a growing root to be subjected to asymmetric chemical treatment. Scale bar, 100 µm. Reproduced from Stanley et al. (2018b) with permission from Wiley-Blackwell Publishing Ltd. (D) Microfabricated root hair growth chambers. *A. thaliana* roots grow through 200 µm deep channels, whereas root hairs grow through 20 µm deep chambers. Scale bar, 200 µm. Adapted from Aufrecht et al. (2017) with permission from MyJove Corp.

High spatiotemporal resolution imaging is an essential technique for studying cellular and subcellular dynamics. A plant root in a microfluidics-based root growth chamber is grown in a microchannel whose height is adjusted such that the root remains in a specific focal plane (**Fig. 2**). This design circumvents z-drift issues that arise in time-lapse imaging of horizontally mounted roots, which typically bend in response to gravistimulation. Microfluidic channels designed to culture Arabidopsis roots are usually prepared using a photolithography technique (Qin et al. 2010). This method, however, is not suitable for creating channel heights > ∼500 µm, as required for observing roots of many other model and crop plants. As an alternative, a 3D-printed replica mold was recently used to prepare 10-mm-deep PDMS channels to study root cells in *Brachypodium* and analyze gene expression under osmotic stress (Khan et al. 2019). In most cases, a straight channel design is chosen for a root growth chamber, but this may not be suitable for observing fibrous root systems. In one case, a multi-chamber design was used to accommodate the branching roots of *Oryza sativa* in a set of radially oriented, petal-shaped chambers (Chai et al. 2019) (**Fig. 2A**). This device also functions as a bioassay platform to investigate the effects of a drought environment (simulated as osmotic stress) on root development by introducing different concentrations of polyethylene glycol (PEG) into each chamber.

Another notable advantage of using microfluidic chambers instead of conventional agar plates is the ability to precisely control the chemical environment around a growing root. Since liquid medium in a microchannel can be manipulated rapidly in space and time, several studies have been conducted to investigate the responses of a growing root to changes in the chemical environment (Grossmann et al. 2011, **Protocol**Grossmann et al. 2012, Busch et al. 2012). For example, a study using a vertically mounted RootChip integrated with pressure-actuated microvalves (**Fig. 2B**) revealed that the rate of Arabidopsis root growth rapidly dropped (within 30 sec) or recovered (within 2 min) in the presence or the absence of auxin, respectively (Fendrych et al. 2018). Since the transcription and translation of auxin-induced genes upon exposure to this phytohormone require much more time (Hoyle and Ish-Horowicz 2013), the authors suggested that auxin-mediated root growth might be regulated by a non-transcriptional branch of the auxin-signaling pathway. While on-chip valve systems enable rapid modulation, they also demand considerable efforts regarding the experimental setup. When slower rates of liquid exchange or rather constant conditions are desired, simple one-inlet-one-outlet chamber designs may be sufficient. As an example, the RootChip-8S, consisting of multiple parallel channels for horizontal (or vertical) mounting, has been used to track the polarization of the cellular growth machinery during root hair formation via confocal spinning disk microscopy (Denninger et al. 2019, **Protocol**Guichard et al. 2020).

Besides chemically treating an entire root, local chemical stimulation on a specifically defined part of a root has been performed by taking advantage of laminar flow conditions, a major characteristic of a microfluidic environment. The first example of this strategy was reported in 2010 in a study demonstrating that local exposure of a small segment of an Arabidopsis root to synthetic auxin led to local root hair growth at the treated site (Meier et al. 2010). Instead of flowing reagent to the growing root in a perpendicular direction, liquid medium can be introduced parallel to the root axis, forming an asymmetric chemical gradient (Stanley et al. 2018b; **Protocol**Stanley et al. 2018a) (**Fig. 2C**). In this perfusion device, the Arabidopsis root is immobilized in the center of a channel using an array of micro-pillars, allowing chemical treatment to be performed on one side of the growing root under laminar flow conditions. Molecular diffusion still occurs at the interface of two laminar liquid streams, and the width of the interdiffusion zone increases as the flow proceeds downstream (Kamholz and Yager 2001). However, when a plant root is confined in a channel whose height is comparable to the diameter of the root, such interdiffusion is likely to be minimized. Because the root serves as a wall between the two flow streams, it facilitates a distinct asymmetry in a chemical environment along the growing root. The ability to generate external chemical heterogeneity on a specific region of a growing root allows us to investigate whether each root cell coordinates its own response to the outer environment or whether the whole root systematically responds to the environment. Using the dual-flow RootChip, root hair growth was examined under asymmetric treatment along a growing root with NaCl, phosphate and a PEG solution simulating drought conditions. These studies revealed significant differences in root hair growth between two environments, suggesting that each root hair cell responds to its surroundings in an autonomous manner.

Root hairs of vascular plants are common subjects of research to understand how a plant cell determines its specification, coordinates its position and regulates the machinery to change its shape (Gilroy and Jones 2000). However, high-resolution imaging of cellular dynamics in growing root hairs on a typical agar plate is challenging, as root hairs grow rapidly and elongate not only on the x–y plane but in the z direction as well. To circumvent this issue, microfabrication techniques have been employed to constrain growing root hairs to a given focal plane under a microscope. The key technical requirement is to create channels of two different heights to accommodate both the main root and root hairs inside the device. This has been accomplished by fabricating a two-layer PDMS mold using a photomask aligner (Aufrecht et al. 2017) (**Fig. 2D**) and by preparing two PDMS layers with different channel depths separately and binding them together (**Protocol**Yanagisawa et al. 2018). On the former platform, a 63× oil objective lens was used to capture root hair organelles, and a higher magnification (e.g. a 100× oil objective lens) could potentially be obtained using an inverted microscope because the root hairs grow directly on the surface of a cover slip. Using the same design, growing root hairs were successfully treated with a reagent without disrupting their positions, an approach that could be advantageous for studying the effects of chemical treatment on growing root hairs in real time.

### Examining root–microbe interactions

Plant roots secrete various metabolites and proteins into their surroundings, forming a rich and complex chemical environment around the roots (Sasse et al. 2018). The carbon sources released from the roots sustain bacterial communities; in return, the plant derives several benefits from the bacteria, such as uptake of mineral nutrients (Jacoby et al. 2017), protection from pathogen attack (Berendsen et al. 2018), and increased adaptability to environmental stress (Rolli et al. 2015). Understanding this interdependent relationship is crucial for improving plant health and crop yields, and there has been growing interest in investigating the interactions between roots and microorganisms at high spatiotemporal resolution. Co-culturing Arabidopsis roots and root-pathogenic nematodes in a microfluidic chamber were first reported in 2011, demonstrating the feasibility of this technique for phenotypic characterization through live imaging of their interactions (Parashar and Pandey 2011).

A breakthrough method published in 2017, which visualized fluorescently labeled root–bacteria interactions in a microfluidic environment (Massalha et al. 2017; **Protocol**: Massalha et al. 2019), has received considerable attention from the microbiology community (Poole 2017). Using fluorescently labeled *Bacillus subtilis* and *Escherichia coli*, the time course of bacterial movement toward a specific area of the root surface and the process of bacterial colonization were successfully observed. The device was mounted on a microscope, and each microchannel was connected to a syringe pump. This experimental setup allowed liquid medium to be introduced or extracted. In a different study, it has been reported that the timing and types of molecules secreted from the root vary along its length (Pini et al. 2017). Thus, having temporal control over the introduction of bacteria around a growing root is a powerful approach for studying the dynamics of cell-to-cell communication in the rhizosphere. In addition, the cultured liquid medium inside the microchannel can subsequently be transferred off-chip to analyze its composition using techniques such as mass spectrometry.

The microfluidic environment offers the possibility for long-term imaging studies. For instance, in one study, the spatial distribution and rate of colonization of bacteria along an Arabidopsis root were successfully tracked for 4 days in a microfluidic device (**Protocol**Aufrecht et al. 2018). In an alternative approach, the authors of the EcoFAB technique presented a simple PDMS device made from a 3D-printed mold, with sufficient space for developing root systems and the inclusion of sand or soil particles to co-cultivate plant roots and microbes in a more natural microenvironment (**Protocol**Gao et al. 2018). Another group cultured a root of a *Populus tremuloides* (aspen tree) seedling in a microfluidic device for more than 1 month (Noirot-Gros et al. 2020). Although these microfluidic devices have some advantages over conventional Petri dishes, the small volume of liquid inside a microchannel can be an issue for long-term observation, as it is difficult to maintain adequate nutrition for root growth. While a perfusion system can be used to exchange liquid medium in a microchannel, the flowing medium might disturb the migration of microbes. In such cases, the pillar structures described in **Fig. 2C**, which were originally designed to maintain the position of a root in a channel, can be used as pockets to trap bacteria around the root (Stanley et al. 2018b); this strategy could be effective for long-term imaging of root–microorganism interactions.

### The moss *Physcomitrium patens*

The moss *Physcomitrium patens* (also known as *Physcomitrella patens*) has become a popular model plant due to its ease of genetic manipulation, rapid growth and outstanding regeneration ability. Unlike flowering plants, *P. patens* remains haploid for most of its lifecycle, and endogenous gene targeting via homologous recombination is highly efficient in *P. patens* (Cove et al. 2006). The combination of these factors makes *P. patens* an attractive model for studying cellular and subcellular processes in an evolutionary context, often by high-resolution live-cell imaging.

**Fig. 3:**
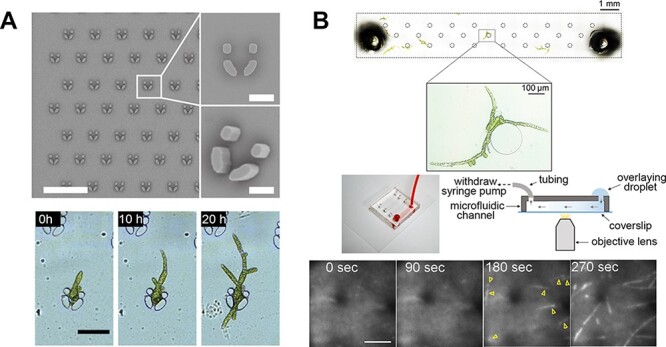
Microfluidic growth chambers for imaging the moss *Physcomitrella patens*. (A) PDMS-based traps to isolate single protoplasts and monitor progeny from single cells. Scale bar, 500 µm (top left image), 40 µm (top right images) and 100 µm (bottom image). Adapted from Sakai et al. (2019) with permission under a Creative Commons Attribution 4.0 International License. (B) Microfluidic chamber for performing a microtubule re-growth assay using HILO sheet imaging combined with liquid perfusion. Scale bar, 5 µm. Adapted from Kozgunova and Goshima, (2019) under a Creative Commons Attribution 4.0 International License.

Traditionally, two live-cell imaging techniques have been used to observe *P. patens*. In the first method, a piece of plant tissue is placed between two coverslips or an agar block and coverslip and immediately imaged. Samples can be prepared rapidly using this method, but cell conditions are variable and growth does not always proceed normally (Kozgunova and Goshima, 2019, Bascom et al. 2016). In the second method, a small piece of protonemal tissue composed of tip-growing cells is cultured in the thin layer of agar on a glass-bottom dish for several days prior to observation. This system allows cells to be propagated as a single-cell layer close to the coverslip, facilitating high-resolution imaging (Yamada et al. 2016). While this method works well for healthy protonemal cells, it is challenging to observe plants with growth defects or during later stages of development, such as gametophore development (leafy shoots). To address these problems, a microfluidic device was designed for long-term culture and imaging of *P. patens* (Bascom et al. 2016). This device is composed of a central circular 30-µm-deep chamber and radial flow control channels. When moss cells were provided with medium that was replaced on a regular basis, they grew inside the microdevice for several weeks with growth rates comparable to samples cultured on solid agar medium. Due to the spatial constraints within the growth chamber, the majority of the cells, including young gametophores, were amenable to confocal microscopy. Furthermore, the authors obtained high-resolution images and quantitative data of tip growth speed for the slow-growing *Δbrk1* mutant that would be challenging to obtain by traditional imaging methods. Another microfluidic platform was developed to monitor single-cell fate in *P. patens* over a long period of time (Sakai et al. 2019). In this study, the authors used an array of pillar traps to capture *P. patens* protoplasts or spores and optimized growth conditions to facilitate protoplast survival and regeneration (**Fig. 3A**). The authors successfully visualized the first cell division and cell wall regeneration and tracked the progeny of single cells at high resolution for 40 h. This technique could potentially be further developed to incorporate more complex flow-control systems for manual cell sorting, single-cell sequencing or mutant isolation based on an observed phenotype.

Microfluidic devices for *P. patens* have also been developed for specialized types of microscopy, such as highly inclined and laminated optical (HILO) sheet imaging (Kozgunova and Goshima, 2019, Leong et al. 2018). HILO imaging is a high-resolution microscopy technique related to the total internal reflection fluorescence technique that allows the observation of subcellular events on the cell surface (e.g. cytoskeleton or plasma membrane protein dynamics). In a pioneering study, researchers used shallow channels to increase the visible cell surface for HILO imaging (Leong et al. 2018). In a subsequent study, using an optimized channel depth for HILO imaging, cell viability was more stable in the microfluidic device compared to samples under coverslips. The same microfluidic device was suitable for drug treatment and robust washout conducted in parallel with high-resolution imaging, for example, to observe microtubule nucleation in vivo (**Fig. 3B**) (Kozgunova and Goshima, 2019). Although Bascom et al. also used a microfluidic device to introduce a drug, washout efficiency was not tested (Bascom et al. 2016). Finally, a microfluidic device was successfully used for long-term (48 h) HILO imaging to visualize changes in the cytoskeleton during cell reprogramming, which was previously inaccessible using samples under coverslips (Kozgunova and Goshima, 2019).

## Microfluidics to Study Pollen Tubes

Sexual reproduction in flowering plants involves pollination, pollen tube growth and double fertilization (of the egg cell and central cell), leading to seed development. The pollen tube is a highly specialized structure composed of a vegetative cell forming the tube and a generative cell giving rise to two sperm cells, which are transported within that tube. The pollen tube is capable of rapid elongation by tip growth and responds to environmental cues by altering its growth direction, facilitating its singular biological function: finding and fertilizing a female gametophyte. Because of their central role in seed set, both the mechanical and cellular aspects of pollen tube growth have received considerable interest over the years.

### Assay of mechanical force in pollen tube cells

Pollen tubes must grow through the tissues of the pistil, passing through multiple mechanical obstacles along the way. The growth and invasive force of the pollen tube depend on the delicate balance between cell wall stiffness at the tip of the cell and turgor pressure, which drives its elongation (Cameron and Geitmann 2018). In vitro pollen tube assays for phenotyping or studying cell biological processes were traditionally performed by growing the cells in Petri dishes filled with liquid or agar-based medium (Reimann et al. 2020). However, unless a direction trigger is provided, pollen tube growth occurs in arbitrary directions, which makes the conventional imaging approach low throughput, as many pollen tubes change the focal plane and are difficult to follow over time. Furthermore, it is challenging (if not impossible) to imitate the mechanical obstacles that pollen tubes encounter in vivo using the conventional method. To circumvent these problems, a microfluidic platform known as TipChip was developed to provide a base concept for a large variety of pollen tube assays (Agudelo et al. 2013a, 2013b; **Protocol**Jo et al. 2017). Since pollen tubes cannot be manipulated and placed with forceps like plant roots, the TipChip for pollen tube growth had to be designed such that the microscopic pollen grains could be positioned at desired locations purely using laminar fluid flow (Ghanbari et al. 2014). The TipChip was also used to study the pollen tube’s behavior when meeting physical obstacles. In a microchannel design fabricated with repeated microgaps narrowing gradually from 17 to 10 µm, elongating tubes were guided to squeeze through these openings (Sanati Nezhad et al. 2013). While the relatively large pollen tubes of *Camellia* (typical width 17 µm) could navigate a modest constriction by changing their own shape or deforming the elastic PDMS material, narrower openings below 10 µm caused the tubes to stall. After successful passage through a constriction, the tubes often burst. A similar study using *Torenia fournieri* pollen tubes (Yanagisawa et al. 2017) revealed that the narrower tubes from this species were more malleable than those of *Camellia* as they were able to overcome extremely tight gaps (1 µm wide and 4 µm high). The same study also demonstrated that both the vegetative nucleus and the sperm cells were successfully carried through the narrow gaps, suggesting that these pollen tubes would have been capable of fertilization. Finally, the authors showed that the ability to navigate narrow openings is present in other tip-growing cells as well, including root hairs (*A. thaliana*) and moss protonemata (*P. patens*), suggesting that all tip-growing plant cells can modify their shapes to overcome mechanical obstacles (Yanagisawa et al. 2017).

**Fig. 4:**
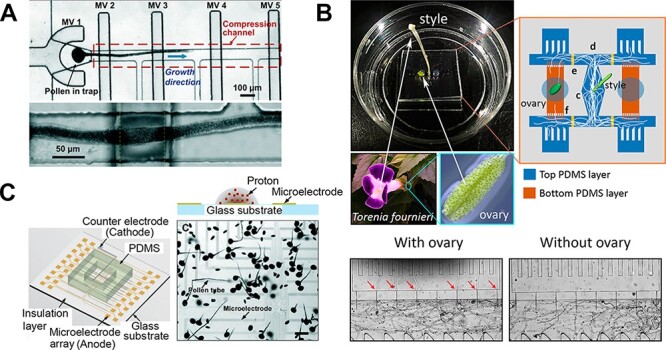
Microfluidics-based pollen tube bioassay methods. (A) Device containing microvalves to compress *Lilium longiflorum* pollen tubes. Adapted from Hu et al. (2017a) with permission from The Royal Society of Chemistry. (B) Micro-slit channel filter device used for a chemotropism assay of *Torenia fournieri* pollen tubes (top image), and a comparison of pollen tubes passing through micro-slit channels (4 and 16 μm gaps) in the presence and absence of an ovary (bottom image). Scale bar, 200 µm. Adapted from Yanagisawa and Higashiyama (2018) with permission from AIP Publishing LLC. (C) Microelectrode array device to generate a local pH gradient. DC voltages are applied to each electrode independently. Electrolysis of water on the surfaces of the electrodes causes local changes in pH; their effects on pollen tube growth can be observed under a microscope. Scale bar, 250 µm. Adapted from Hu et al. (2017b) with permission from The Royal Society of Chemistry.

The micro-gap device was also used to measure the dilating force that the pollen tube exerts to navigate constricting environments. Based on the elastic deformation behavior of PDMS, the dilating force was calculated and deduced to correspond to an internal cell pressure of 0.15 MPa (Sanati Nezhad et al. 2013). The actual quantification of the invasive force of the pollen tube required a very different design approach. A complex, five-layered microfluidic device was designed to present the elongating tube with a calibrated cantilever so as to measure the maximal force it was able to exert (Ghanbari et al. 2018). Another microfluidic platform combined with a microelectromechanical systems (MEMS)-based force sensor was used to examine the correlation between the force exerted by the pollen tube and the corresponding changes in growth direction (Burri et al. 2018). Various compression systems have also been integrated into microfluidic platforms to directly assess the mechanical properties of growing pollen tubes, such as cell wall stiffness and turgor pressure. An early study used a microfluidic platform consisting of multiple parallel channels to unify the direction and focal plane of pollen tube growth (Shamsudhin et al. 2016). Using this platform combined with cellular force microscopy, high-throughput micro-indentation experiments were successfully performed on *Lilium longiflorum* pollen tubes. Another approach was to incorporate a deformable PDMS membrane operated by valves inside the microdevice (**Fig. 4A**) (Hu et al. 2017a). In this device, *L. longiflorum* pollen tubes that passed through the channel were softly compressed by the PDMS membrane and then subjected to osmotic stress or excessive pressure to determine the pollen tube stretch ratio. Using finite modeling, the mechanical properties of the pollen tube were determined, such as cell wall stiffness and turgor pressure. Drawbacks of this platform are the low throughput of experimentation, as it only accommodates a single pollen tube, as well as the disruptive procedure required to measure the stretch ratio. These issues were addressed by the development of a micro-robotic system featuring two indenters (Burri et al. 2019). By sequentially applying compression with a plate-indenter and micro-indentation with a tip-indenter, the authors recorded force and displacement in multiple *L. longiflorum* pollen tubes. This dual-indentation system represents a non-invasive method to measure the mechanical parameters of the pollen tubes and monitor how they change over time in individual cells.

### Pollen tube guidance assay

The main role of pollen tubes in flowering plants is to deliver non-motile sperm cells to the ovules for fertilization. Plant ovules produce species-specific molecules that trigger the reorientation of growing pollen tubes to attract them (Márton et al. 2005, Okuda et al. 2009, Takeuchi and Higashiyama 2012, Zhong et al. 2019). Therefore, the mechanism behind gametophytic pollen tube guidance is thought to play a key role in generating reproductive isolation in plants. Chemotropism in pollen tubes is often studied using a semi-*in vitro* assay in which a dissected pistil and ovules are co-cultured on agarose medium in a Petri dish (Kurihara et al. 2013), and pollen tube growth behaviors such as attraction to ovules are assessed under a microscope. Although this simple approach reveals the overall chemotropism of pollen tubes, quantitatively evaluating chemoattracted pollen tubes is quite challenging due to their wavy growth pattern. Several microfluidic devices have been designed to address this issue, including devices with T-shaped or cross-shaped microchannels that confine plant ovules to a pre-assigned channel (Yetisen et al. 2011, Horade et al. 2013, Sato et al. 2015). In these systems, pollen tubes that have entered a particular channel are considered to be chemoattracted and can easily be counted. While these devices provide quantitative data, such as the rate of pollen tube attraction toward ovules, not all pollen tubes are capable of responding to attractants (Mizukami et al. 2016). In this situation, some pollen tubes will likely elongate in any direction regardless of the presence of ovules. To prevent these randomly growing pollen tubes from reaching the ovules, a microfluidic channel filter was developed for *T. fournieri* (**Fig. 4B**) (Yanagisawa and Higashiyama 2018). An array of narrow channels in this system allows chemoattractant molecules to diffuse through, resulting in the generation of a concentration gradient in the assay area. When the width of the filter was 2 µm, which is considerably narrower than the diameter of a pollen tube (∼8 µm), chemoattracted pollen tubes were still able to penetrate through such small spaces. Depending on the size of the filter, 80–100% of the randomly growing pollen tubes were prevented from reaching the ovules; hence, truly chemoattracted pollen tubes could be extracted among populations by employing this method.

While ovules have been used to assay gametophytic pollen tube guidance in these devices, there is a need to develop methods to assess the activity of attractants that trigger pollen tube guidance. One such approach is to manually place a gelatin bead containing purified attractant near the tip of a growing pollen tube using a micro-manipulator system (Okuda et al. 2009). A concentration gradient of the chemical is quickly created around the bead, and the behavior of the growing pollen tube with regard to the presence of this gradient can be examined under a microscope. Although this method is well established, such a manual approach inevitably restricts the throughput of the assay, and the concentration gradient of the attractant cannot be controlled. Various microfluidics-based concentration gradient generators have been reported in the literature (Ahmed et al. 2010), and the TipChip has been successfully adapted to expose growing pollen tubes to an extremely sharp concentration gradient (Sanati Nezhad et al. 2014a). This approach requires careful control of laminar flow but allows dynamic tuning of the position of the gradient to follow the moving and bending pollen tube tip. Thus, it may provide an important tool to test the activity of pollen tube attractants or repellants.

### Microfluidics-based electrotropism assay

Electrotropism, the directional growth of a cell guided by an electric field, has been investigated in pollen tubes of various species. This type of research is motivated by the finding that the pollen tube itself drives an electrical current through the cell due to the local uptake of cations such as Ca^2+^ at the growing tip (Weisenseel et al. 1975). In this situation, when an external electric field is present around a pollen tube, its endogenous field (created by the imbalance of charged ions along the tube) may be affected, often resulting in changes in its growth direction. Since measurable voltages can be detected along a style in vivo (Spanjers 1981), it is reasonable to expect that electrotropism is involved in directional pollen tube growth in certain regions of the style. Although several in vitro studies have been conducted to detect electrotropism in pollen tubes (Wang et al. 1989, Nakamura et al. 1991, Bou Daher and Geitmann 2011), the interpretation of the experimental outcomes is not straightforward because applying voltages to growth medium immediately changes the conditions of the medium itself. For instance, when a DC (direct current) voltage is applied to the growth medium, electrolysis occurs on the surfaces of electrodes, which will alter the pH of the medium if it is not buffered. The changes in medium temperature induced by Joule heating are also a concern. Even under constant voltage, the degree of increase in temperature varies depending on the volume, type (liquid or solid) and conductivity of the medium. Under these circumstances, the results of *in vitro* electrotropism assays are certain to be inconsistent. Microfabrication techniques can be used to address some of these issues. For instance, by depositing thin metal layers onto a glass slide, electrodes can be patterned inside a microfluidic chamber. The effect of Joule heating is almost negligible in a microfluidic channel due to its large surface area relative to liquid volume, which facilitates rapid heat dissipation (Elvira et al. 2013). Also, pollen grains can be precisely placed between the two electrodes, which minimizes the potential variation in experimental outcomes arising from the random positioning of cells in a growth chamber (Agudelo et al. 2016).

In another study, researchers took advantage of the electrolysis of water to demonstrate how the local change in pH around the tip of a pollen tube would affect its growth behaviors (**Fig. 4C**) (Hu et al. 2017b). Upon applying a DC voltage, a pH gradient formed between the electrodes in the microfluidic chamber and the cytoplasmic pH at the tip of a growing pollen tube decreased from ∼7.0 to ∼5.7. As a consequence, distinct cellular behaviors such as arresting, bursting or reorienting away from the electrode were observed. Cell wall acidification can trigger cell wall softening via the activation of cell wall hydrolases (Bosch and Hepler 2005). This softening might lead to an imbalance between internal turgor pressure and stiffness of the apical cell wall, in turn leading to changes in tube growth behavior. The ability to generate a local pH gradient only at the tip of a growing pollen tube should allow the functions of protons and cellular growth to be investigated more precisely, which are difficult to observe using a Petri dish or any type of bulk analysis.

## Future Perspectives

Looking back on 10 years of development in plant-on-a-chip technology, microfluidic techniques have become widely adopted by the plant science community. An increasing number of research articles in this field have been presented by researchers in biological sciences, indicating that more devices are now being designed, created and operated by plant biologists. While photolithography is primarily used to create PDMS molds, 3D-printed PDMS molds have already been employed to create root culture chambers (Chai et al. 2019, Khan et al. 2019). Due to the improving resolution of 3D printers and lower instrument costs, 3D printing will likely become a mainstream approach for the fabrication of PDMS molds (Bhattacharjee et al. 2016), which will further lower the bar to the use of microfluidic tools by plant biologists. Microfabricated chambers are currently in great demand, especially for live-cell imaging. Even a simple, straight microfluidic channel helps researchers keep specimens such as roots, root hairs, pollen tubes or moss protonemata on a single focal plane under a microscope while exchanging growth medium using a perfusion system.

Besides live-cell imaging, there is growing interest in using droplet-based microfluidics for single-cell (protoplast) analysis. By introducing flow streams of cell culture medium and oil-containing surfactant into a microchannel, single cells can be encapsulated into each droplet. Several studies using this technique have been reported, such as single-cell transcriptome analysis of Arabidopsis root protoplasts (Ryu et al. 2019) and the examination of gene expression levels driven by a heat-inducible promoter in individual *Marchantia* protoplasts (Yu et al. 2018). Since commercial microfluidic systems are now widely available, performing single-cell analysis via droplet-based microfluidic technology is no longer technically burdensome. However, few on-chip molecular studies have been performed despite the strong interest in studying cell-to-cell communication in plants. Microfluidics originally emerged as a chemical analysis platform. Various chemical analysis methods (e.g. separations, detection and quantification of molecules) have been developed during its 30-year history (Convery and Gadegaard 2019). While the pressure-driven flow-based perfusion system is dominant in current plant-on-a-chip applications, electro-osmotic flow allows the flow of a liquid to be precisely manipulated in space and time and has therefore been used extensively for molecular analysis in microfluidic devices (Wang et al. 2009). Another way to generate liquid flow in microchannels involves the use of capillary force, which does not require any external equipment for its control (Juncker et al. 2002). The use of this type of autonomous flow could potentially lead to the development of a novel bioassay to assess the responses of plant cells to specific chemicals without the need for manual operations. These well-studied microfluidic techniques have not yet been exploited to study plant cells. We anticipate that strong collaborations between plant biologists and microfluidics researchers will lead to the development of such molecular analysis tools in the future. Looking at the expanding applications of microfluidics, such as drought monitoring through stomatal openings on leaves (Koman et al. 2017), gravisensing in plants by visualizing statolith movement (Bérut et al. 2018) and cytoskeletal organization studies in microfabricated wells (Durand-Smet et al. 2020), there is no doubt that microfluidics will play more diverse roles in plant cell biology research in the future.

## Supplementary Material

pcab067_Supp
